# Inhibitory Effects of Enalaprilat on Rat Cardiac Fibroblast Proliferation via ROS/P38MAPK/TGF-β_1_ Signaling Pathway

**DOI:** 10.3390/molecules17032738

**Published:** 2012-03-06

**Authors:** Min Yu, Yang Zheng, Hong-Xia Sun, Du-Juan Yu

**Affiliations:** 1 Department of Cardiology, First Clinic Hospital Of Jilin University, Changchun 130021, China; Email: 543328673@qq.com (M.Y.); 502311634@qq.com (D.-J.Y.); 2 Department of Cardiology, Affiliated Hospital of Beihua University, Jilin 132011, China; 3 Department of Pharmacology, College of Pharmacy, Beihua University, Jilin 132013, China

**Keywords:** enalaprilat, angiotensin II, cardiac fibroblast, reactive oxygen species, p38 mitogen activated protein kinase, transforming growth factor-β_1_

## Abstract

Enalaprilat (Ena.), an angiotensin II (Ang II) converting enzyme inhibitor (ACEI), can produce some therapeutic effects on hypertension, ventricular hypertrophy and myocardial remodeling in clinic, but its precise mechanism, especially its signaling pathways remain elusive. In this study, cardiac fibroblasts (CFb) was isolated by the trypsin digestion method; a BrdU proliferation assay was adopted to determine cell proliferation; an immunofluorescence assay was used to measure intracellular reactive oxygen species (ROS); immunocytochemistry staining and Western blotting assay were used to detect phosphorylated p38 mitogen activated protein kinase (p-p38MAPK) and transforming growth factor-β_1_ (TGF-β_1_) protein expression, respectively. The results showed that Ang II (10^–7^ M) stimulated the cardiac fibroblast proliferation which was inhibited by NAC (an antioxidant), SB203580 (a p38MAPK inhibitor) or enalaprilat; Ang II caused an burst of intracellular ROS level within thirty minutes, an increase in p-p38MAPK (3.6-fold of that in the control group), as well as an elevation of TGF-β_1_ meantime; NAC, an antioxidant, and enalaprilat treatment attenuated cardiac fibroblast proliferation induced by Ang II and decreased ROS and p-p38MAPK protein levels in rat cardiac fibroblast; SB203580 lowered TGF-β_1_ protein expression in rats’ CFb in a dose-dependent manner. It could be concluded that enalaprilat can inhibit the cardiac fibroblast proliferation induced by Ang II via blocking ROS/P38MAPK/TGF-β_1_ signaling pathways and the study provides a theoretical proof for the application of ACEIs in treating myocardial fibrosis and discovering the primary mechanism through which ACEIs inhibit CFb proliferation.

## 1. Introduction

Myocardial fibrosis is indicated by cardiac hypertrophy and cardiac fibroblast proliferation, together with increased collagen synthesis and changed ingredients, resulting in myocardial stiffness, ventricular systolic and diastolic dysfunction, heart failure or even sudden death. Ang II plays an essential role in stimulating myocardial fibrosis by binding to Ang II type 1 receptor (AT_1_) to stimulate several intracellular signaling cascades [1]. p38MAPK, one of the mitogen activated protein kinase (MAPK) is sensitive to oxidative stress. Ang II can induce ROS generation via oxidative stress reactions, which in turn, activate the p38MAPK signal pathway and both ROS and p38MAPK molecules are involved in myocardial fibrosis formation [2]. It has been reported that Ang II can activate p38MAPK signaling cascades and increase TGF-β_1_ expression by a positive feedback regulatory mechanism, which may facilitate diabetic nephropathy development in renal fibrosis [3]. Recent studies have also showed that p38MAPK and TGF-β_1_ are involved in diabetic nephropathy progression via mutual interactions during the process of secretion of fibronectin in glomerular mesangial cell [4,5]. Enalaprilat can produce definite effects on the treatment of hypertension, ventricular hypertrophy and myocardial remodeling. Many reports have demonstrated that enalaprilat can decrease myocardial collagen deposition dramatically and reverse left hypertrophy and myocardial fibrosis in early stage of spontaneously hypertensive rats by inhibition of the rennin- angiotensin-aldosterone system (RAAS) activity and promotion of bradykinin generation [6]. However, its precise mechanism, especially signaling transduction pathways, remain to be clarified. The purpose of this study was to determine whether the inhibitory effect of enalaprilat, an angiotensin converting enzyme, on CFb proliferation is linked to blocking of ROS-P38MAPK-TGF-β_1_ cascades induced by Ang II.

## 2. Results and Discussion

### 2.1. Effect of Enalaprilat on Cardiac Fibroblast Proliferation

**Figure 1:**
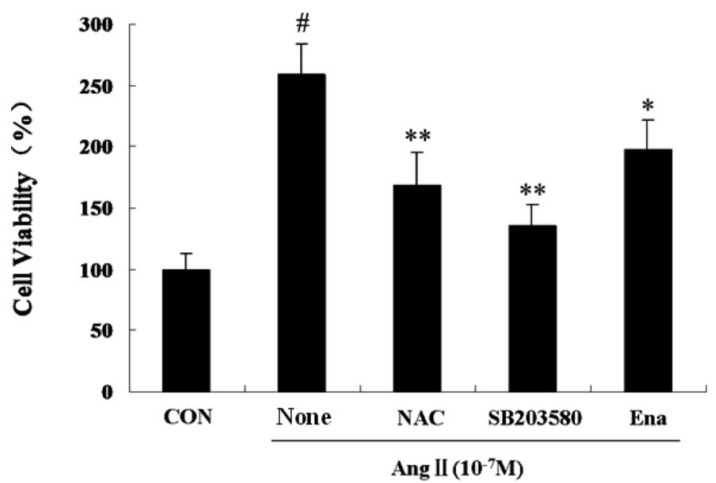
Influence of Ang II on rat cardiac fibroblast proliferation as measured by BrdU incorporation. Cells were grown in the absence or presence of Ang II (10^−7^ M; *Ang II*) and coincubated with the ACE inhibitor enalaprilat (10^−6^ M, *Ena.*), antioxidant NAC (10^−2^ M, 30 minutes prior to AngII administration), p38MAPK inhibitor, SB203580 (10^−7^ M, 30 minutes prior to AngIIadministration) for 24 hours. Bars represent the DNA synthesis as measured by BrdU incorporation with SEM. Activity is shown relative to cells treated with vehicle alone (*C*). Data are presented as mean ± S.E.M. ^#^ *P* < 0.001 *vs.* Control; * *P* < 0.05, ** *P* <0.01 *vs.* Ang II.

The influences of each treatment group on CFb viability continued for 24 hours, Ang II induced a marked CFb proliferation, which was significantly inhibited by co-treatment with antioxidant NAC, p38MAPK inhibitor SB203580 or enalaprilat (Figure 1).

### 2.2. Effect of Enalaprilat on Intracellular Reactive Oxygen Species Generation

**Figure 2:**
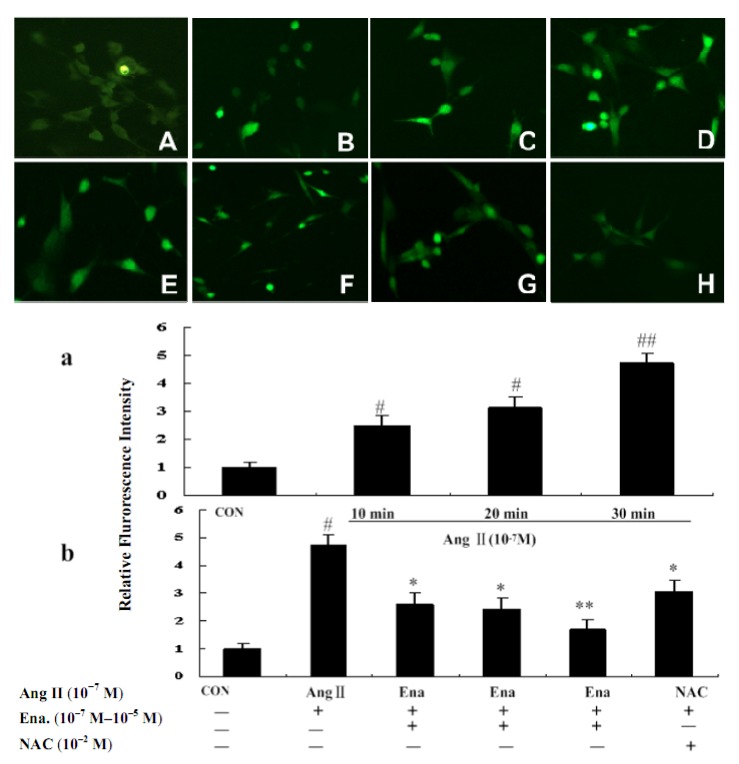
Effects of Ena. and antioxidant NAC on ROS intensity of cardiac fibroblasts in fluorescence microscope (200×). CFb were cultured in Dulbecco’s Modified Eagle Medium containing Ang II (10^−7^ M) for 10 minutes, 20 minutes and 30 minutes, and treated with enalaprilat (10^−7^ M, 10^−6^ M, and 10^−5^ M) for 30 minutes. CFb were labeled with 2',7'-dichlorofluorescein diacetate for 30 minutes and ROS generation was analyzed by fluorescence detection with fluorescent microscopy at 200×. Upper part: **A**:CFb were cultured in Dulbecco’s Modified Eagle Medium without Ang II; **B**: CFb were stimulated with Ang II for 10 min; **C**: CFb were stimulated with Ang II for 20 min; **D**: CFb were stimulated with Ang II for30 minutes; **E**–**G**: CFb were stimulated with Ang II and treated with Ena.(10^–7^ M, 10^–6^ M, and 10^–5^ M respectively) for 30 minutes; **H**: CFb were stimulated with Ang II and treated with NAC (10^–2^ M, 30 minutes prior to Ang IIadministration) for 30 minutes. Lower part: **a** and **b**: Statistic representations are indicated as bars in involved group. Data are presented as mean ± S.E.M. ^#^ *P* < 0.05, ^##^ *P* < 0.01 *vs.* control; * *P* < 0.05, ** *P* < 0.01 *vs.* Ang II group.

Recent studies have demonstrated that growth factors stimulate ROS production in various cell types. To determine whether Ang II induces intracellular ROS in cardiac fibroblasts, we measured the intracellular oxidative levels using the hydroperoxide-sensitive fluorophore 2',7'-dichlorofluorescein diacetate by immunofluorescent staining. Cells treated with Ang II had significantly higher fluorescence intensity than cells treated with vehicle (Figure 2). The rise in 2',7'-dichlorofluorescein diacetate fluorescence was partially blocked by enalaprilat. Pretreatment with a flavoprotein containing the NADH/NADPH oxidase inhibitor NAC reduced Ang II stimulated fluorescence intensity. These findings suggested that Ang II triggered an elevation of ROS and enalaprilat can reduce ROS levels through an anti-oxidative route.

As a signal transduction molecule, ROS changes quickly, therefore we observed the data of consecutive alterations after effecting Ang II at 10 min intervals within 30 mins. The results showed that ROS of the treated group was elevated gradually after 10 min, peaked at 30 min, and then, dropped (data not shown) compared with that of the control group (*P* < 0.05 or *P* < 0.01). ROS was decreased with enalaprilat treatment in a dose-dependent manner. Enalaprilat and NAC inhibited ROS increases within 30 minutes, showing that Ang II promoted ROS generation by oxidation, while NAC reduced ROS generation through clearing the oxidative products by anti-oxidation. Enalaprilat lowered the level of ROS associated with CFb proliferation induced by Ang II, as shown in Figure 2.

### 2.3. Protein Analysis of Phosphorylated p38MAPK by Western Blot

**Figure 3:**
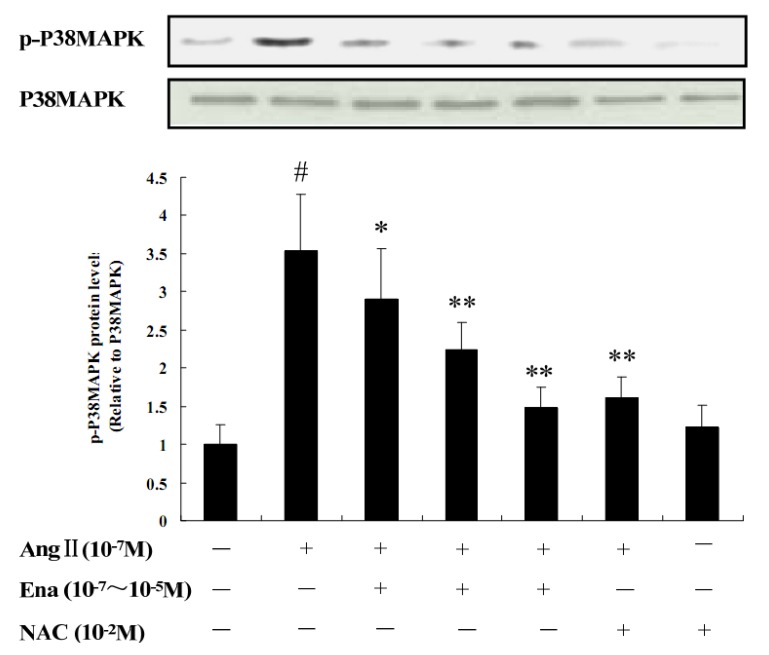
The Effect of AngII, Ena. and antioxidant NAC on p-p38MAPK protein expression by Western blotting in CFb. AngII (10^−7^ M), Ena. (10^−7^ M, 10^−6^ M, 10^−5^ M), NAC (10^–2^ M, 30 minutes prior to Ang II administration). Representative blots are shown. Data are shown as relative level to the control group (p-p38MAPK protein expression level / p38MAPK = 1), Data are expressed as mean ± S.E.M. (n = 8). ^#^ *P* < 0.05 *vs.* control; * *P* < 0.05, ** *P* < 0.01 *vs.* Ang II.

It can be seen from Figure 3 that p-p38MAPK protein expression was significantly increased in the Ang II stimulated group compared with that in the control group (*P* < 0.05); different concentrations of enalaprilat could reduce p-p38MAPK protein expression to certain levels compared with that in Ang II stimulated group, Ena. 10^−^^7^ group (*P* < 0.05), Ena. 10^−6^ and Ena. 10^−5^ group (*P* < 0.01); compared with that in Ang II group, p-p38MAPK protein expression in Ang II + NAC group (*P* < 0.01), which represented Ang II activated the expression of p-p38MAPK through oxidative stress way, NAC inhibited p-p38MAPK expression by antioxidant pathway. Compared with the control and Ang II groups, the NAC treated group had no difference, which meant NAC itself had no effect on the expression of p-p38MAPK.

### 2.4. Effect of Enalaprilat on p38 MAPK Phosphorylation in CFb

**Figure 4:**
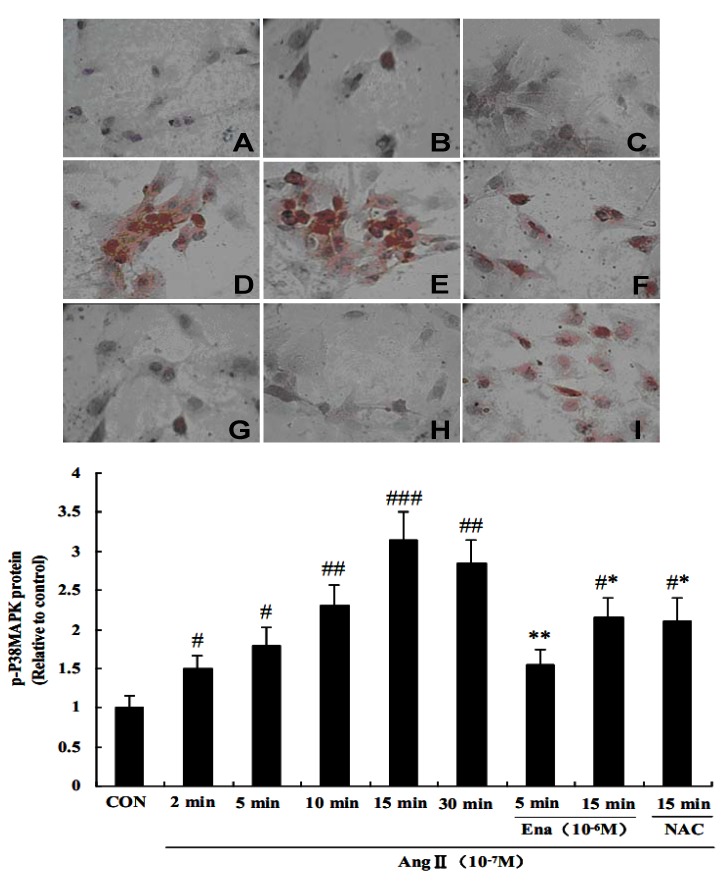
Effects of Ang II, Ena., NAC on p-p38MAPK nuclear translocation. **A**: p-p38MAPK nuclear translocation was observed under inverted microscope (400×) with immunocytochemistry staining; **B**: Statistic representation of p-p38MAPK nuclear translocation is indicated as bars in the involved group. CFb were cultured in Dulbecco’s Modified Eagle Medium containing Ang II (10^–7^ M). Brown staining in cytoplasm and nuclei represented protein expression of p-p38MAPK. Figure 4A, **A**: Control group; **B**–**F**: Ang II induced for 2 minutes–30 minutes; **G**–**H**: Ena. (10^–6^ M) treated for 5 and 15 minutes; **I**: NAC (10^–2^ M, 30 minutes prior to AngII administration) for 15 min. ^#^ *P* < 0.05, ^##^ *P* < 0.01, ^###^ *P* < 0.001 *vs.* control; * *P* < 0.05, ** *P* < 0.01 *vs.* Ang II.

The expression of phosphorylated p38MAPK (p-p38MAPK) was significantly induced by Ang II. p-p38MAPK protein expression was seen in the cytoplasm at 2 min, while it was seen in nuclei at 5 minutes, which showed that Ang II induced the nuclear translocation of p-p38MAPK in CFb beginning within 5 minutes after exposure to Ang II. The p-p38MAPK nuclear staining was the densest at 15 minutes (Figure 4 **A**–**E**), which stood for the quantity of p-p38MAPK entering the nucleus was the most at this time (*P* < 0.001), and it began to decrease at 30 min, compared with the control group (*P* < 0.05 or *P* < 0.01). p-p38MAPK expression began to decline after Ang II treatment for 30 minutes. Enalaprilat could inhibit p38MAPK nuclear translocation for 5 minutes and 15 minutes, so did the NAC group for 15 min, compared with control and the corresponding time of Ang II treated group (*P* < 0.05. Figure 4 **A**–**G**, **H**, **I**).

### 2.5. Effect of Enalaprilat on TGF-β_1_ Protein Expression

**Figure 5:**
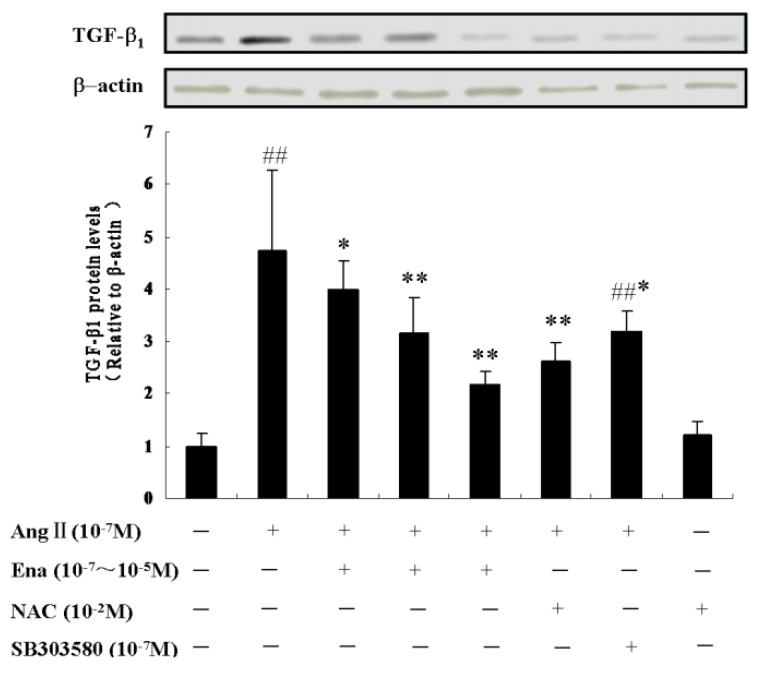
Effect of Ena., NAC and SB203580 on TGF-β_1_ protein expression of CFb by Western Blot, Ang II (10^−7^ M), Ena. (10^−7^ M, 10^−6^ M, 10^−5^ M), NAC (10^−2^ M, 30 minutes prior to Ang II administration), SB203580 (10^−7^ M, 30 minutes prior to Ang II administration). The black bars show statistic representations of TGF-β_1_ protein level, data are shown as relative to the control group in which TGF-β_1_ protein level = 1.0. ^##^ *P* < 0.01 *vs.* control; * *P* < 0.05, ** *P* < 0.01 *vs.* Ang II group.

Determination of TGF-β_1_ protein is according to the basic principle of the internal reference control of both the general semi-quantitative experiments. To achieve the semi-quantitative measurement of the expression differences in TGF-β_1_ protein expression between different samples, it is necessary that the number of cells between different samples be the same so that the amount of differences can reflect expression differences between cells. To do so, the expression of a protein generally expressed in various cells, such as the β-actin was used as internal reference to normalize TGF-β_1_ protein expression levels. It can be seen from Figure 5 that TGF-β_1_ protein expression began to rise after Ang II stimulation in CFb compared with the control group (*P* < 0.01).

Enalaprilat at different doses reduced TGF-β_1_ protein expression compared with the Ang II group (*P* < 0.05 or *P* < 0.01); The TGF-β_1_ expression dropped in the Ang II + NAC group compared with the Ang II treated group (*P* < 0.01), which indicated that Ang II promoted TGF-β_1_ protein expression through ROS signal pathways, the antioxidant NAC inhibited the increase of TGF-β_1_, but could not decrease it to normal levels completely, which showed that NAC partially blocked the TGF-β_1_ ascending stimulated by Ang II. TGF-β_1_ expression in Ang II + SB203580 group dropped compared with the Ang II group (*P*< 0.05), which demonstrated that Ang II caused TGF-β_1_ protein expression by acting on the p38MAPK signaling pathway, and compared with control group (*P* < 0.01), which meant SB203580 partially suppressed p38MAPK pathway and moved forward a single step to block TGF-β_1_ from increasing. NAC itself had no difference compared with control group, indicating that it had no effect on TGF-β_1_ expression.

Recent reports have shown that Ang II stimulates membrane-bound nicotinamide adenine dinucleotide phosphate (NADPH) oxidase, which generates ROS in cardiac fibroblasts [7]. Previous studies have shown that Ang II enhances ROS synthesis and release via stimulating NADPH oxidase [8,9]. ROS is regarded as a pivotal factor in the occurrence of cardiovascular diseases involving the activation of the rennin-angiotensin-aldosterone system and allopurinol (a xanthine oxidase inhibitor) alleviates the left ventricular remodelling by decreasing the collagen deposition in extracellular matrix and improves the cardiac function following myocardial infarction by reducing ROS generation, indicating a significant role of ROS in myocardial fibrosis [10]. ROS generation induced by an oxidative stress reaction was involved in hamster lung fibroblast proliferation [11]. ROS played a role of the second messenger to act on p38MAPK in the signal transduction cascades, and inhibition of p38MAPK by SB203580 or scavenging ROS by NAC reduced lung fibroblast proliferation [11] and Ang II-induced vascular fibrosis through fibroblast phenotypic differentiation to myofibroblast via ROS-p38MAPK pathways in vascular smooth muscle cells [12]. As a molecule in the signaling transduction pathway activated by stress, p38MAPK participated in the renal fibrosis and the myocardial fibrosis, and the inhibition of p38MAPK could improve the left ventricular function and attenuate the ventricular remodeling following myocardial infarction in rats [13]. NADPH oxidase inhibitors and catalase could block p38MAPK phosphorylation stimulated by Ang II [14]. These results suggest that ROS and p38MAPK are involved in the myocardial fibrosis induced by Ang II. Our data demonstrated that enalaprilat inhibited CFb proliferation induced by Ang II, reduced ROS generation and blocked ROS-mediated phosphorylated p38MAPK nuclear translocation and subsequent p-p38MAPK protein expression in CFb in response to Ang II. These data suggest that the inhibitory effect of enalaprilat on CFb proliferation may be mediated by suppressing the activation of ROS-p38MAPK signal pathways.

ROS could be activated by p38 mitogen-activated protein kinase, Janus kinase, signal transducer and activator of transcription pathways to induce the gene transcription of nuclear factor-κB (NF-κB) and activator protein-1 (AP-1) [15]. p38MAPK could bind to activator protein-1 to induce its own auto-phosphorylation and become activated [16], which might enhance TGF-β_1_ expression, aggravate the glomerular and tubular interstitial cell hypertrophy and increase the extracellular matrix synthesis mediated by its downstream response medium–connective tissue growth factor (CTGF) to promote the progression of diabetic nephropathy [17]. SB203580 could suppress the synthesis of α_1_ (I) procollagen and its gene expression induced by TGF-β_1_ in CFb, improve the deposition of type I collagen and the expression of α-smooth muscle actin, reduce type χ collagen and fibronectin contents in myocardium, which was mediated by p38MAPK signaling pathway after myocardial infarction [13]. These results indicate that p38MAPK is closely related to the collagen deposition and the fibroblasts phenotypic differentiation in extracellular matrix. Gruden showed that p38MAPK independently induced TGF-β_1_ and fibronectin protein expressions [17], In turn, TGF-β_1_ contributed to maintaining p38 MAP kinase activation, which perpetuated fibronectin accumulation. Research discovered that there were distinct interactions between TGF-β_1_ and p38MAPK [18,19]. These results have confirmed that the interactions between p38MAPK and TGF-β_1_ are key mechanisms involved in the progression and persistence of the fibrosis.

It has been verified that Ang II induces p38MAPK via ROS-p38MAPK signal cascades and then p38MAPK was translocated into the nucleus and turned into phosphorylated p38MAPK after being activated to induce its target gene generation through stimulating its downstream factor, such as activator protein-1, nuclear factor-κB and other transcription factors which were extremely sensitive to ROS [21,22]. It is worth noting that there was an activator protein-1 consensus sequence in TGF-β_1_ gene promoter region, a large number of TGF-β_1_ would be expressed in CFb after being bound with activator protein-1, which induced the phenotypic switch of CFb to myofibroblast to stimulate CFb proliferation and promote extracellular matrix synthesis on one hand, and induce the secretion of collagen fibers on the other hand [21,22,23,24,25]. It is demonstrated that the gene promoter region of procollagen α_1_ (I) contained the TGF-β_1_ response element which located between the transcription starting site of −174~84 bp [26] and procollagen α_2_ (I) TGF-β_1_ response element which situated at the site of −246~300 bp [27]. TGF-β_1_ binding with the corresponding TGF-β_1_ response element activated procollagen gene expressions, such as typeⅠcollagen, type Ⅲ collagen and fibronectin, leading to myocardial fibrosis. Our data confirmed that enalaprilat reduced p-p38MAPK and TGF-β_1_ expressions in CFb in a dose-dependent manner. The results indicate that enalaprilat inhibits the production of TGF-β_1_ through blocking p38MAPK signal pathways to weaken the positive feedback regulatory mechanism between p38MAPK and TGF-β_1_, which may prevent the occurrence of myocardial fibrosis accordingly. 

Little has been reported about the molecular mechanisms through which enalaprilat can inhibit myocardial fibrosis, especially in signaling transduction pathways [28] and it is not clear whether enalaprilat suppresses CFb proliferation and reduces extracellular matrix deposition via inhibiting ROS-p38MAPK-TGF-β_1_ signaling transduction pathways. The results presented in this study suggested that p38MAPK was activated rapidly after Ang II induction, p-p38MAPK protein expression was seen in cytoplasm at 2 minutes, the nuclear translocation was seen at 5 minutes, p-p38MAPK protein activity reached the peak at 15 minutes, and then began to decrease at 30 minutes; NAC could inhibit the up-regulation of p-p38MAPK protein expression, which illustrated that Ang II could induce myocardial fibrosis through the ROS-p38MAPK signal pathways; p-p38MAPK expression was down-regulated after being treated with enalaprilat at different times, which was also in accordance with the results from Western blot assay analysis and indicated that enalaprilat inhibited CFb proliferation via inhibition of ROS-p38MAPK pathways. In this study, the results from Western blot assay verified that SB203580, p38MAPK inhibitor and enalaprilat reduced TGF-β_1_ protein expression.

## 3. Experimental

### 3.1. Chemicals

NAC (N-acetyl-L-cysteine, antioxidant) was purchased from Sigma (St. Louis, MO, USA). All MAPK inhibitor, SB203580 was purchased from Cell Signaling (Beverly, MA, USA). All chemicals were obtained from Merck (Darmstadt, Germany) and Sigma Chemicals, (Deisenhofen, Germany) if not otherwise specified.

### 3.2. Cell Isolation

The isolation of cardiac fibroblasts from neonatal rats was performed according to a modified protocol as described previously. Briefly, the hearts of 1–3 day-old rats (Wistar-Kyoto) were isolated and digested with 10 mL of Spinner-solution (in mM): NaCl 116, KCl 5.3, NaH_2_PO_4_ 8, NaHCO_3_ 22.6, HEPES 10, D-glucose 5, pH 7.4, containing 0.1% collagenase (Cytogen, Berlin, Germany) for 10 min at 37 °C in eight consecutive steps. After each digestion, the medium containing the suspended cells was removed and an equal volume of Spinner/collagenase solution was added. The cardiac cell suspension was mixed with an equal volume of Ham’s F10 (Gibco BRL, Eggenstein, Germany) supplemented with 10% fetal calf serum (FCS; c.c.pro, Hamburg, Germany) and 25 mg/mL 71 Gentamycin (Gibco BRL), and stored at 48 °C. The cells were centrifuged at 1 × 10^6^ g for 5 minutes, and the cell pellets were re-suspended in 20 mL of Ham’s F10 supplemented with 10% FCS and plated on culture dishes. After 75 minutes, the medium, which contained the cardiomyocyte fraction of the digested tissue, was removed. The dishes were gently rinsed three times to remove the remaining cardiomyocyte. The culture medium for cardiac fibroblasts was changed for Dulbecco’s Modified Eagle Medium (DMEM; Gibco BRL) supplemented with 20% FCS and 25 μg/mL gentamycin. Purity of cardiac fibroblast culture (>97%) was assessed with repeated differential plating, microscopic evaluation and immunostaining.

### 3.3. Proliferation Assay

After an incubation in serum-free Dulbecco’s Modified Eagle Medium for 24 h, the cells (25,000 cells per well of a 96-microtiter plate), passages 2 ± 3, were stimulated with Ang II and coincubated with an antioxidant agent NAC and a p38MAPK inhibitor, SB203580 (Bachem Biochemica GmbH, Heidelberg, Germany), Ena. (the bioactive metabolite of enalapril). Cellular proliferation was assessed with 5-bromo-2'-deoxyuridine (BrdU, 40 μg/mL) incorporation during the last 4 hours of the 24 hours incubation period, and then, fixed in 4% paraformaldehyde for 30 minutes at room temperature. Following the fixation, the cells were incubated with 50% formaldehyde in 2× SSC at 65 °C for 30 min and with 2 N HCl at 37 °C for 10 min. After the incubation with 0.1 M boric acid at room temperature for 3 minutes, the cells were rinsed with PBS and blocked with 1% bovine serum albumin at room temperature for 1 hours, followed by the incubation with an anti-BrdU antibody overnight at 4 °C. Cells were then incubated with a FITC-conjugated secondary antibody to visualize BrdU positive labeled cells with a colorimetric immunoassay according to the manufacturer’s guidelines (Boehringer Mannheim, Mannheim, Germany) as described before. The extinctions were measured at 450 nm in an ELISA plate reader. All values consist of an n = 9.

### 3.4. Immunofluorescence Assay of Intracellular ROS

Intracellular ROS production was measured by using the fluorescent dye 2',7'-dichlorofluorescein diacetate (DCF-DA) (Molecular Probes, Eugene, OR, USA) with the ACAS interactive laser cytometer (Meridian Instruments, Inc., Okemos, MI, USA), as described previously (Cheng *et al.*, 1999). A 10 mM stock solution of 2',7'-dichlorofluorescein diacetate was prepared in ethanol on a daily basis and diluted to a final concentration of 10 uM just before the experiments were conducted. Cardiac fibroblasts were preincubated with 10 uM 2',7'-dichlorofluorescein diacetate in Dulbecco’s Modified Eagle Medium for 30 minutes at 37 °C before treatment. After exposure to the dye, the cells were rinsed with Tyrode’s solution. The cells were maintained in Tyrode’s solution and examined by using the laser cytometer at 37 °C. Excitation of 2',7'-dichlorofluorescein diacetate was achieved by using the 488-nm line of a 20-mW argon-ion laser. The emission above 515 nm was quantitated from two-dimensional scans generated by using a 1-um laser beam and an X-Y scanning stage to obtain a fluorescence value from single cells. To provide a valid comparison, the same acquisition parameters were used for all observations. The quantification of the levels of 2',7'-dichlorofluorescein diacetate fluorescence was assessed on a relative scale from 0 to 4,000 units. Baseline values from unstimulated cells were used as control values in the comparison with Ang II-stimulated cells. Values represent mean ± S.E.M. of 2',7'-dichlorofluorescein diacetate from 20 randomly selected cells for each experiment in the five investigations.

### 3.5. Immunocytochemistry

Pure cardiac fibroblasts were grown in 24-well plates and cultured until the confluence formed. After 24 h incubation in serum-free Dulbecco’s Modified Eagle Medium, the cells were fixed with 4% paraformaldehyde in phosphate buffer (10 mM, pH 7.4) after Ang II (10^−7^ M, for 2 minutes, 5 minutes, 10 minutes, 15 minutes, 30 minutes) and Ena. (10^−6^ M, for 5 minutes and 15 minutes) treatment respectively. After being washed with PBS, the cells were blocked in 0.5% BSA in PBS, incubated with the primary antibody, p-p38MAPK (1:500, Chemicon, Temecula, CA, USA), and then diluted in PBS containing 0.1% BSA. Incubations were performed for 2 h at 37 °C. After several washes in PBS, the wells were incubated with biotinylated secondary antibodies (LSAB kit, DAKO, Carpinteria, CA, USA) for 1 h at 37 °C. The staining was visualized by the biotin–streptavidin peroxidase method (LSAB kit, DAKO). 

### 3.6. Western Blot Assay

Neonatal rat cardiac fibroblasts, passages 2 ± 3, were starved for 24 hours in serum-free medium. After exposure to Ang II (10^−7^ M), Enalaprilat (10^−7^ M, 10^−6^ M, 10^−5^ M), NAC (10 mM, 30 minutes pre-incubation with Ang II administration), SB203580 (10^-7^M, 30 minutes pre-incubation with Ang II adminstration), the cells were lysed in 0.5 mL of the following buffer: 50 mM NaCl, 20mM Tris (pH 7.4), 50 mM NaF, 50 mM EDTA, 20 mM sodium pyrophosphate (Na_4_P_2_O_7_), 1 mM sodium orthovanadate (Na_3_VO_4_), 1% Triton X-100, 1 mM PMSF, 0.6 mg/mL leupeptin and 10 μg/mL aprotinin. The protein content was measured with a standard Bradford assay. Total cell lysates (40 μg/lane) were analyzed with sodium dodecyl sulphate polyacrylamide electrophoresis (SDS ± PAGE) and electrophoretically transferred to a nitrocellulose membrane. Immunoblotting was performed with monoclonal antibodies against (p38MAPK, anti-phospho-p38, TGF-β_1_) (1:1,000), all groups were treated for 24 hours. (all antibodies: Santa Cruz Biotechnology Inc., Heidelberg, Germany). A horseradish peroxidase-labelled goat anti-rabbit IgG antibody (Amersham, Buckinghamshire, UK, 1:5000) was used as the secondary antibody and the densitometric analysis was performed on an Epson GT 8000 scanner by using the analysis software ScanPack (Biometra, Goëttingen, Germany).

### 3.7. Statistical Analysis

All values are mean ± S.E.M. The significance of differences among mean values was determined by ANOVA. The statistical comparison of the control group with treated group was performed with Fisher’s multiple comparison tests. The accepted level of significance was *P* < 0.05.

## 4. Conclusion

The results described above prove that enalaprilat can block the nuclear translocation of p-p38MAPK and its phosphorylation induced by Ang II and reduce TGF-β_1_ generation to resist CFb proliferation through diminishing ROS generation, thereby reverse the occurrence of myocardial fibrosis, implying that enalaprilat inhibits CFb proliferation and extracellular matrix deposition through blocking ROS-p38MAPK-TGF-β_1_ signaling transduction pathways.
